# Development and initial testing of an in vitro model simulating class II furcation defects

**DOI:** 10.1002/cre2.346

**Published:** 2020-12-06

**Authors:** Jørgen Hugo, Odd Carsten Koldsland, Anne Merete Aass, Hanna Tiainen

**Affiliations:** ^1^ Department of Periodontology, Institute of Clinical Dentistry University of Oslo Oslo Norway; ^2^ Department of Biomaterials, Institute of Clinical Dentistry University of Oslo Oslo Norway

**Keywords:** biofilm, furcation defect, in vitro model, porcine, root surface roughness

## Abstract

**Objective:**

To compare surface topography of porcine and human root dentin and to develop a new in vitro model for class II furcation defects. The hypothesis for this study was that porcine mandible blocks can function as a model for class II furcation defects.

**Background:**

Treatment of mandibular class II furcation defects is unpredictable. There is a need for in vitro models to investigate new treatment methods.

**Methods:**

A model to investigate the surface topography of porcine and human root dentin was developed and the two tissues compared by SEM imaging and profilometer. A novel method for studying class II furcation defects was then tested. Blocks of porcine mandibles with molar 3 were prepared. Buccal class II furcation defects were created. The furcation area was isolated and bioluminescent *Staphylococcus epidermidis* Xen43 was used to form a biofilm in the furcation area to test the functionality of the novel furcation model.

**Results:**

Micromechanical damage caused by debridement on porcine and human root dentin showed similar pattern. No significant difference in the surface morphological parameters was observed between the corresponding porcine and human samples. The model allowed for assessment of the root surface inside the furcation area. While the number of viable bacteria in the furcation following debridement could be quantified, no significant difference between the treatment groups was detected, likely due to bacterial colonization within the periodontal ligament space.

**Conclusion:**

Porcine and human root dentin show similar surface topography following surface debridement. Porcine mandible blocks can function as a model for class II furcation defects. However, further development and refinement of the novel in vitro model is warranted.

## 1 INTRODUCTION

Furcation defects resulting from bone loss around multi‐rooted teeth are common in periodontally compromised patients and present a challenging treatment setting for the clinicians (Svardstrom & Wennstrom, 1996). A reduced long‐term prognosis and increased chance of tooth loss has been reported for furcation involved teeth (Nibali et al., 2016; Waerhaug, 1980).

The primary treatment goal for furcation involved teeth is to improve the prognosis and survival by adapting the local anatomical conditions to better facilitate plaque control and/or to attempt regeneration of the lost periodontal tissues (Sanz & Giovannoli, 2000). Plaque control is regarded as the determinant factor for success or failure for both regenerative periodontal surgery (Cortellini & Tonetti, 2015) and for the treatment outcome of periodontal disease in general (Axelsson, Nystrom, & Lindhe, 2004; Lindhe, Westfelt, Nyman, Socransky, & Haffajee, 1984). This can be especially challenging around furcation involved teeth, with class II defects generally regarded as the most problematic. Periodontal treatment is performed with or without surgical access depending on the severity of bone loss, local anatomy and accessibility for scaling and root planing (SRP) (Lindhe et al., 1984).

The changes in surface topographical properties following mechanical periodontal treatment may alter the conditions for biofilm renewal (Leknes, Lie, Wikesjo, Bogle, & Selvig, 1994). Host tissue cells may also be affected by the morphological properties of the surface (Hagi et al., 2015; Khosravi, Bahrami, Atabaki, Shokrgozar, & Shokri, 2004). Hence bacterial adhesion and biofilm formation as well as the potential for re‐establishing the periodontal supportive tissues are closely linked to root surface roughness. It is therefore important to evaluate the topographical changes following instrumentation of root surfaces. However, measuring parameters such as root surface roughness can be difficult in a clinical setting. At the same time it is also important to understand how the exposed root surface acts in contact with the oral environment. It is therefore necessary to explore alternative methods to a clinical setting in order to obtain these objectives. The use of extracted teeth in an in vitro model may provide a novel approach for investigating this highly complex biological system. Extracted human teeth are becoming increasingly difficult to obtain for research purposes due to a general increase in oral health as more and more patients keep their teeth for life (Rozier, White, & Slade, 2017). The use of human teeth also require individual patient consent and storage in an approved biobank (Coppola et al., 2019). It is warranted to explore alternatives that possess similar properties that may substitute human samples. To the best of our knowledge, there is no existing model that utilizes extracted teeth, human or porcine, to simulate class II furcation defects in vitro.

The hypothesis for this study was that porcine mandible blocks can function as a model for class II furcation defects. The furcation model is intended to be used for studying mechanical and biological properties of regenerative materials, the effect of periodontal treatment on root dentin and surrounding bone and antimicrobial agents used in the treatment of periodontal disease.

Considering this, the present study was conducted to establish an in vitro model using porcine mandible blocks that resembles the anatomical environment seen around class II furcation involved human teeth in vivo. Such a model needs to fulfill certain criteria and must:

1. Represent a reproducible method to simulate class II furcation defect
2. Show similar root surface morphology as human root dentin
3. Allow for assessment of the root surface inside the furcation
4. Allow for microbiological status to be obtained and assessed

The primary aim of this study was to investigate the use of porcine mandible blocks as a functional in vitro model for class II furcation defects. The secondary aims were (A) to evaluate and compare the effect of surface debridement on surface morphology of porcine and human root dentin and (B) to investigate biofilm formation inside the furcation area using the current model.

## 2 MATERIALS AND METHODS

The current study was designed as an in vitro study.

### 2.1 Porcine furcation model

**FIGURE 1:**
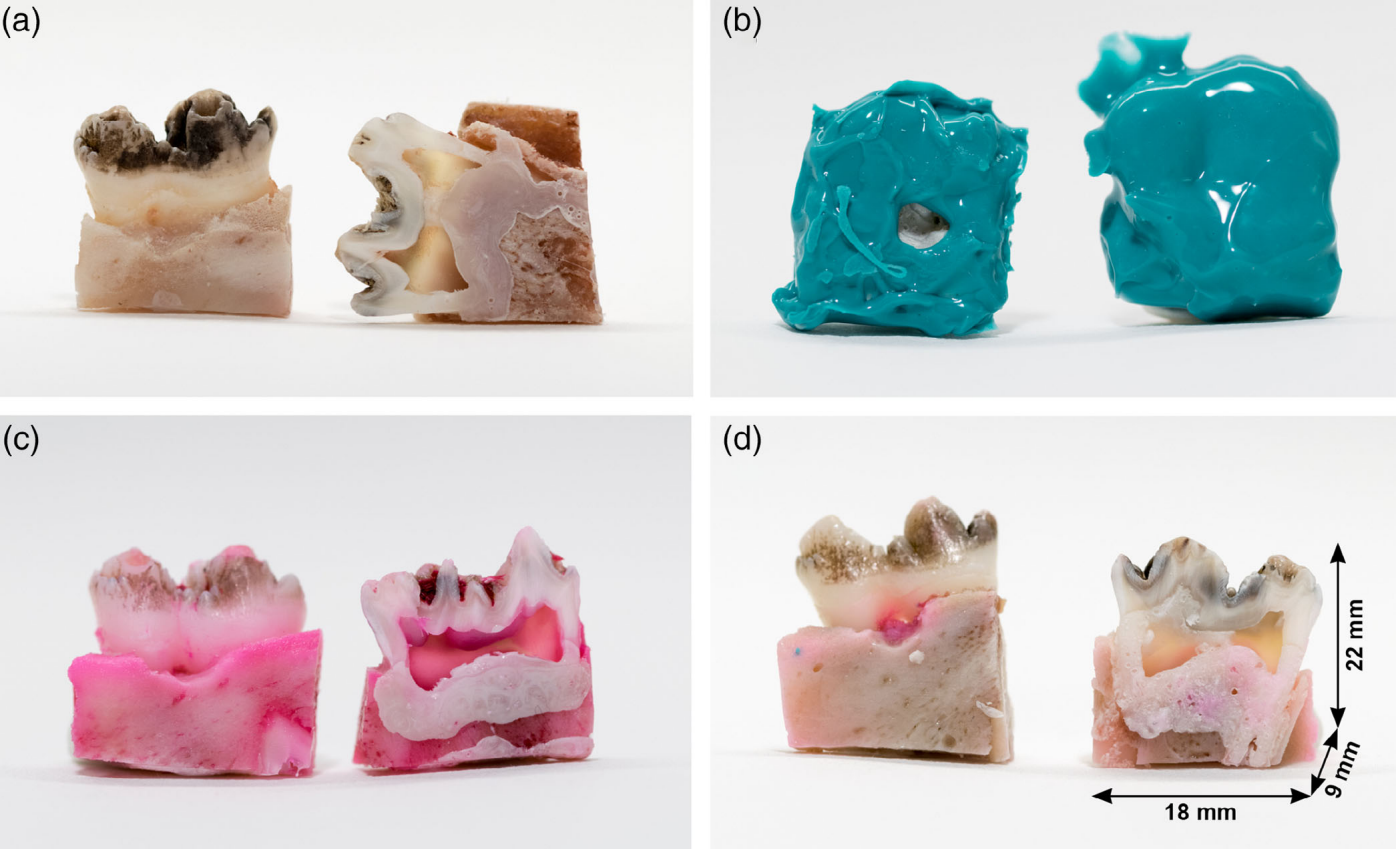
(a) porcine mandible block without silicone or staining (b) porcine mandible block with sealing material prior to staining, (c) porcine mandible block stained with 0.3 mM rhodamin B for 24 h (control) and (d) porcine mandible block after 24 h in staining solution (silicone removed). Average dimensions of the prepared bone blocks are given in (d). Average depth of the created furcation defect was 3 mm

Fresh porcine mandibles with soft tissue left intact were obtained. The jaws were split in the midline and frozen at −20°C pending study start. Removal of the soft tissue was done by hot and cold maceration (Sullivan, 1999). Porcine mandibles were boiled for 3 h in 10 L of water containing 70 ml of enzymatic laundry detergent and left to cool for 48 h. Remaining soft tissue was subsequently removed and the samples were thoroughly rinsed with water to remove any traces of the laundry detergent. The samples were prepared using a diamond blade band saw^1^ so that the back lower molar (M3) and the bone around the tooth remained intact. The bone and tooth were separated 3 mm in order not to damage the root surface during the furcation preparation. Buccal osteotomy was performed using a rosen burr^2^ to create a class II furcation defect with average depth of 3.2 ± 0.3 mm. Bone and tooth were repositioned, and the samples fixed on the opposite side of the furcation using acrylic resin^3^ (Figure 1(a)). To evaluate the possible loss of sealing capacity due to dislocation of the roots from their socket, 10 samples were also prepared without separating the tooth from the bone block as a control. The average dimensions of the final samples were 22 ± 2 mm in height, 18 ± 4 mm in width, and 9 ± 1 mm in depth as measured using a digital caliper (Figure 1(d)). The samples were autoclaved for 40 min at 121°C, transferred to a sterile hood and embedded in silicone impression material^4^, leaving the furcation exposed (Figure 1(b)).

The samples (n = 9) were then imaged by x‐ray microtomography^5^ at 11 μm pixel size using source voltage of 70 kV and a current of 133 μA with a 0.5 mm aluminum filter to optimize contrast. Samples were rotated 180° around their height axis and four absorption images were recorded every 0.40° of rotation. The recorded images were then reconstructed to serial coronal‐oriented tomograms using a 3D cone beam reconstruction algorithm (NRecon^6^). Image analysis of the reconstructed 3D datasets was performed using standard Skyscan analysis software (DataViewer, CTan and CTvox)^††^. Furcation height was measured as the vertical distance from the cementoenamel junction (CEJ) to alveolar bone floor of the artificially created furcation defect, while the furcation depth was measured as the horizontal distance from the buccal root surface to the bottom of the furcation as illustrate in Figure S1. Total volume of the created furcation defect was determined as the volume of missing tissues within the volume delimited by the defect wall and a planar surface extending vertically from the CEJ to the buccal bone surface at the bottom of created bony defect. Selection of the volume of interest for the volumetric analysis is illustrated for a single plane in Figure S2.

To test the silicone seal, test samples were immersed in a staining liquid (0.3 mM rhodamine B) for 24 h and compared to control samples without silicone seal. To further test the sealing properties of the silicone, bacteria were cultivated inside the furcation area and biofilm formation within the isolated furcation area was registered with SEM imaging.^7^

### 2.2 Surface analysis using profilometer

To compare the effect of different root debridement methods on the surface morphology of porcine and human root surfaces, 3D surface morphological parameters were assessed quantitatively on planar dentin surfaces using an optical profilometer.^8^ For this purpose, samples of human and porcine root dentin were prepared by cutting of the root. The human teeth were collected at the dental faculty, University of Oslo and stored in a dark cabinet on a solution of chlorhexidine digluconate in water (1 mg/ml) pending study start. The human teeth were immersed in deionized water for 24 h, then put in an ultrasonic bath containing deionized water for 10 min before they were cut.

Root dentin pieces were fixated to a plastic slide using two‐component epoxy resin.^9^ The slides were then mounted on a grinding machine and polished down to a flat level using p800, p1200 and then p4000 SiC grinding paper.^10^ Curette,^11^ Wærhaug's diamond^12^ and ultrasonic tip^13^ were used to treat separate samples for 30 s. Three different operators performed the treatment (J.H., O.C.K. and A.M.A.). Polished, untreated samples were used as control. Following treatment and drying, samples were scanned using ×50 objective. Each operator treated four samples per treatment group and three non‐overlapping scans (255 × 191 μm^2^) were obtained for each sample (n = 12 per operator; n = 36 when pooled). The use of human teeth in this study was approved by the Regional Committee for Research Ethics, Oslo, Norway (2016/1473 and 2016/402).

### 2.3 Bacterial study

Bacterial stocks of bioluminescent *S. epidermidis* Xen43 were stored at −80°C in 15% glycerol until use. Stocks were used to prepare diluted cultures in tryptic soy broth (TSB) at an initial optical density of 0.05 at 600 nm (OD_600_) as determined spectrophotometrically^14^ before the suspension was cultured for 12 h until OD_600_ 0.4–0.5. The overnight cultures were diluted in TSB 1:50 and cultured for another 12 h to obtain OD_600_ 0.4–0.5. The second overnight cultures were diluted in TSB to obtain OD_600_ = 0.05–0.07 (~1 × 10^7^ cells ml^−1^) and used to inoculate sealed porcine samples. Each sample group was placed in separate standard 6‐well plates.^15^ Subsequently 200 μl of bacterial suspension was added to the furcation area of each sample before incubating at 37°C for 1 h. After 1 h the wells were filled with TSB until the furcation was completely covered. The samples were incubated for 24 h at 37°C to form a biofilm. Samples incubated in TSB without bacteria inoculation in the furcation area were used as negative control to ensure that only the inoculated bacteria were present in the samples. All incubation steps were performed in an aerobic atmosphere. Following 24 h incubation, the samples were either left untreated (control) or the biofilm was removed with curette^***^, Wærhaug's diamond^†††^, or ultrasonic tip^‡‡‡^ for 30 s. All samples were rinsed in phosphate buffered saline (PBS) before and after treatment. The silicone seal was removed post treatment and the bone and teeth separated. Samples were bath sonicated in 1500 μl PBS in individual wells of 12‐well plates for 3 min at 30 kHz with power output of 290 W. Dilution series were prepared (10^−1^ to 10^−7^) in PBS. Negative control samples were not diluted. Subsequently 15 μl of diluted bacteria suspension was plated on tryptic soy agar plates and incubated for 24 h at 37°C before counting the colony forming units (CFU). For each sample 15 μl of 10^−3^ dilution was resuspended in 185 μl of fresh TSB and incubated on 96‐well opaque microplates^16^ sealed with transparent adhesive seals^17^ in a multi‐purpose plate reader^18^ for 18 h while luminescence was measured every 15 min during the entire incubation period.

Samples from each group were fixed in 2.5% glutaraldehyde in 0.1 M Sørensen's phosphate buffer followed by dehydration in graded ethanol series (50%, 70%, 90%, 100%; 10 min each step) and left to dry in ventilation hood overnight. Samples were then sputter coated with gold^19^ before imaging with scanning electron microscope.^20^ For high magnification images of the furcation area, five or more overlapping images taken at different focal depths were merged^21^ to increase the depth of focus in the presented figures.

Each experiment was performed four independent times with three replicates per group for each repetition (n = 12).

### 2.4 Statistical analysis

Statistical analysis was done using SigmaPlot 13.0 (Systat Software Inc). One‐way ANOVA analysis on ranks (Kruskal‐Wallis test) with post hoc Dunn's test was used to compare treatment groups and control. Statistical difference was considered at *p* < 0.05.

## 3 RESULTS

### 3.1 Model setup

The prepared class II furcation defects showed relatively low individual variation in their morphological properties as shown in Figure S3. The average height and depth of the furcation defect were measured from the microCT image section to be 3.1 ± 0.5 mm and 3.4 ± 0.8 mm, respectively, while the average volume of the created furcation defects was 26.8 ± 5.7 mm^3^, including the entire buccal osteomy. The depth measurements corroborate the clinical furcation depth measurements performed using a physical probe (3.2 ± 0.3 mm).

The water sealing properties of the silicone/porcine root dentin interface were investigated by submerging the porcine mandibular blocks isolated with silicone impression material (Figure 1(b)) in 0.3 mM rhodamine B solution for 24 h. The results showed minimal staining outside the unsealed furcation area on the sealed samples compared to samples without silicone seal (Figure 1(c, d)). However, separating the tooth from the bone block revealed slight staining of the root surface below the bone block for some of the silicone‐isolated samples. This was not observed for samples that were prepared without lifting the tooth from the socket during preparation of the furcation defect. When placing 200 μl of staining solution in the exposed furcation area, which mimics the bacteria seeding process, no loss of surface tension in the seeded droplet was observed for these samples during 1 h incubation period prior to immersing the entire sample in the staining solution for 24 h.

**FIGURE 2:**
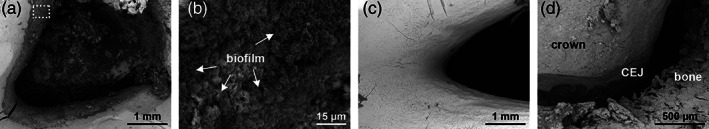
(a) Biofilm formation on porcine root dentin after 24 h incubation (note the well‐defined border between biofilm and clean root surface), (b) higher magnification of area highlighted in A with arrows pointing towards thick layers of bacterial biofilm on the root surface, (c) furcation area after sonication and (d) gap space between the root surface and surrounding bone

The sealing properties observed for water were confirmed by culturing bacteria within the exposed furcation area. A clear and well‐defined biofilm formed in the exposed furcation area following 24 h incubation in aerobic conditions (Figure 2(a, b)). No biofilm formation was observed in the surrounding area covered with sealing material.

The entire biofilm could be detached from the root dentin surface for quantification by bath sonication as no remaining bacteria was observed on samples that were sonicated prior to SEM imaging (Figure 2(c)). The detached bacteria were viable following the bath sonication process as quantified by CFU counting. The SEM imaging of the bone blocks also revealed that a gap was observed between the root surface and the surrounding bone in some of the samples; this gap coincided with the location of the periodontal ligament space on test samples (Figure 2(d)).

### 3.2 Surface morphology

**FIGURE 3:**
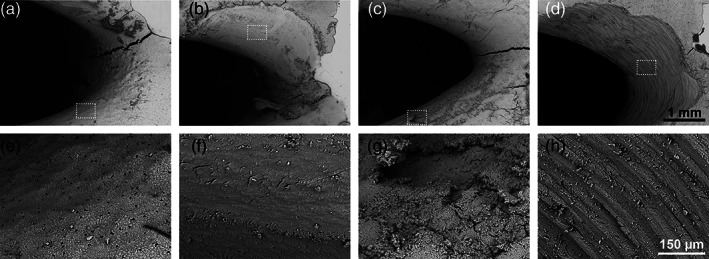
(a, e) porcine furcation area without instrumentation (control), (b, f) porcine furcation area after treatment with curette, (c, g) porcine furcation area after treatment with ultrasonic device and (d, h) porcine furcation area after treatment with Wærhaug's diamond. Bottom row (e–h) shows the areas highlighted in the top row (a–d) at higher magnifications

**FIGURE 4:**
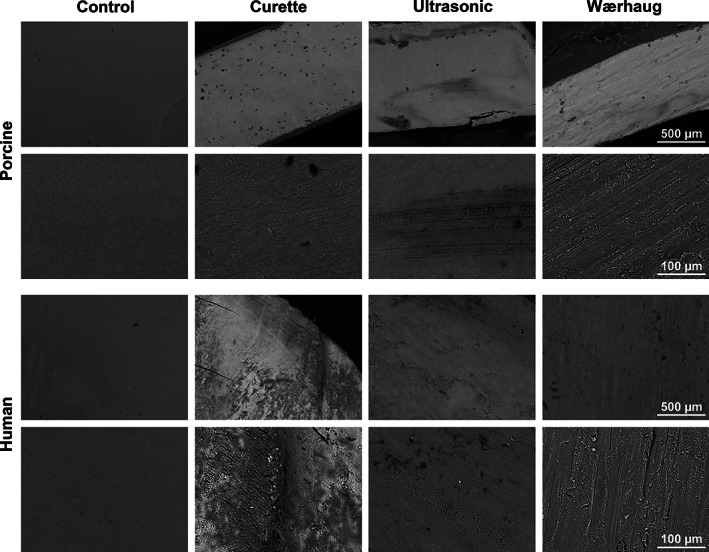
porcine root dentin samples: control (no treatment), treatment with curettes, treatment ultrasonic device and treatment with Wærhaug's diamond. Human root dentin samples: control (no treatment), treatment with curettes, treatment ultrasonic device and treatment with Wærhaug's diamond

Markings on the root dentin surface was observed regardless of the instrument used for root surface debridement. Furcation area treated with a curette generally showed localized scratches in areas where the curette tip had been in contact with the surface, while shallow loss of root dentin substance was observed in areas where the curettes touched the surface at a perpendicular angle (Figure 3(b, f)). The same was observed for samples treated with an ultrasonic tip. However, the application of ultrasonic tip appeared to produce narrower and more well‐defined imprints (Figure 3(c, g)). Wærhaug's diamond consistently left deep, homogenous markings with a substantial loss of root dentin substance (Figure 3(d, h)). Similar topographical surface patterns were observed when applying the same treatment on the planar root dentin samples used for the quantitative analysis of surface morphology (Figure 4). Furthermore, no qualitative difference was observed between the porcine and human samples treated with the same instrument.

**FIGURE 5:**
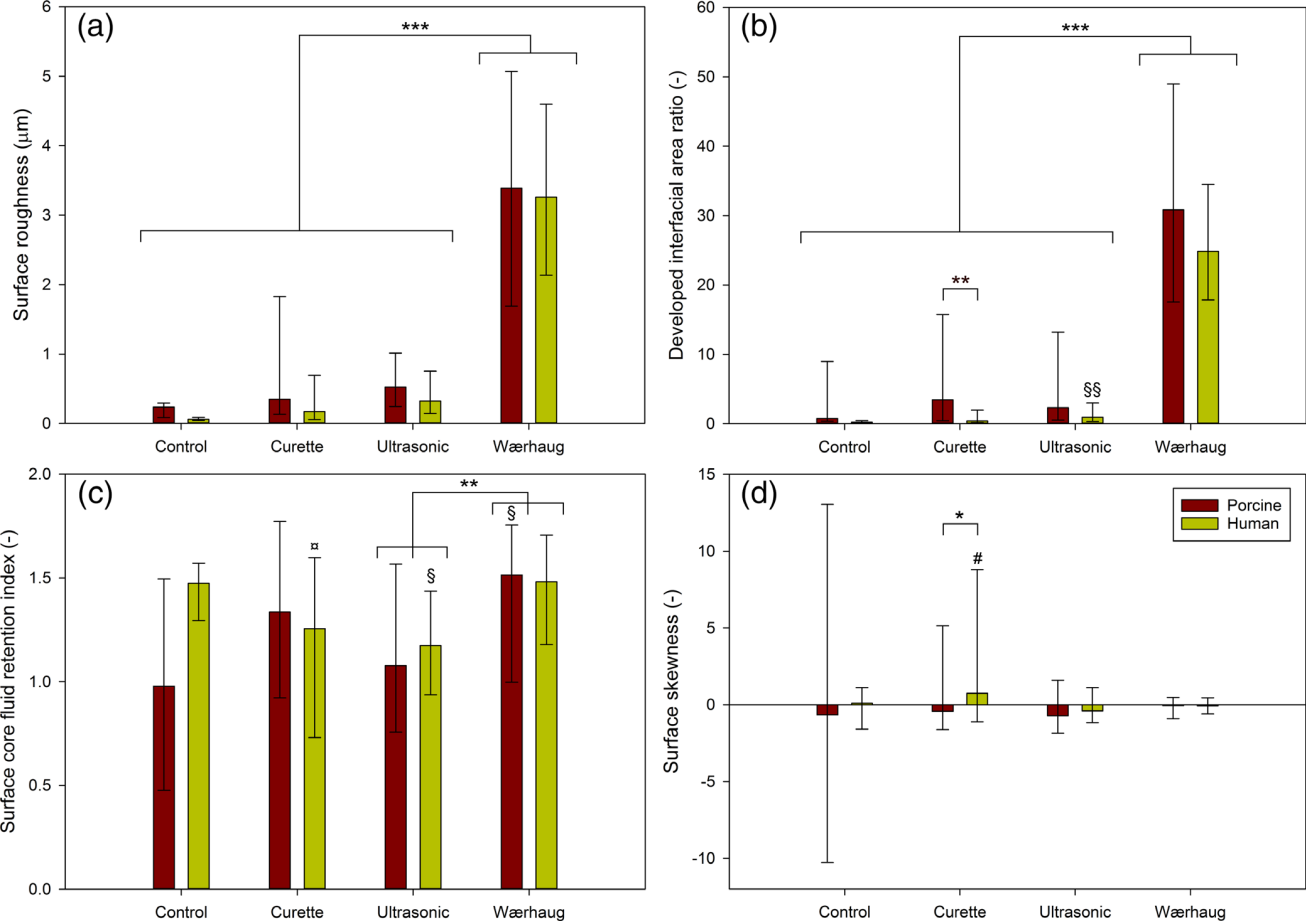
(a) mean values surface roughness (Sa), (b) mean values developed interfacial area ratio (Sdr), (c) surface core fluid retention index (Sci) and (d) mean values surface skewness (Ssk). Symbols: ^*xxx*^ *p* < .001, ^*xx*^ *p* < .01, ^*x*^ *p* < .05, *x* = any symbol; *: against all indicated groups, §: against corresponding control, ¤: against corresponding Wærhaug's diamond, #: against corresponding ultrasonic and Wærhaug's diamond, *n* = 36 for all groups

Changes in surface morphology of both porcine and human root dentin samples were quantified using optical profilometry. As no statistically significant difference between samples treated by different operators were found, all samples were pooled for statistical analysis. The profilometer scans showed a statistically significant increase in the average surface roughness (Sa) for the Wærhaug's diamond treated group as compared to control, curette and ultrasonic tip. The difference between porcine and human samples was not statistically significant. The same was observed for the developed interfacial area ratio (Sdr). The mean Sdr value was slightly higher for the porcine samples, though this difference was not statistically significant. For both porcine and human samples, the Sa and Sdr for control, curette and ultrasonic groups were not significantly different (Figure 5(a, b)).

The surface core fluid retention index (Sci) showed statistical significant difference between the ultrasonic tip and Wærhaug's diamond, the latter showing a higher value. The remaining differences were not statistically significant and there was no difference observed between porcine and human samples (Figure 5(c)).

Surface skewness (Ssk) showed a high variability for control samples on porcine root dentin. There was a significantly higher value for human compared to porcine teeth when the curette was used, otherwise no difference was observed (Figure 5(d)). Overall human and porcine root dentin show very similar properties. The morphological features observed qualitatively using SEM correlated to those produced on the planar 2D root dentine blocks, the results for the quantitative 3D surface morphological analysis obtained on the planar samples was assumed to correspond to the corresponding parameters on the root surface of the 3D furcation defects.

### 3.3 Biofilm formation

**FIGURE 6:**
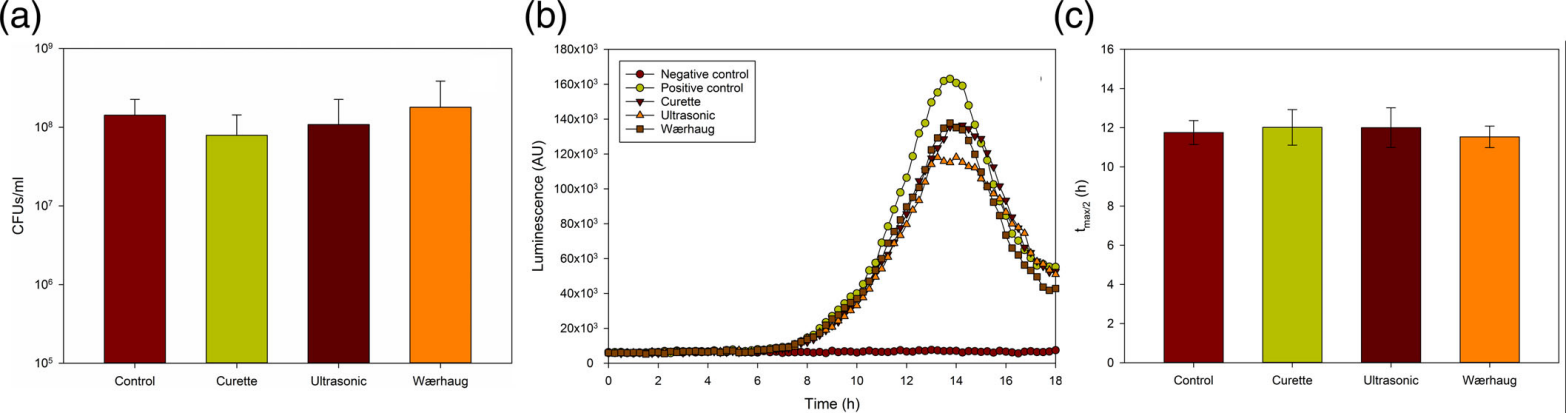
(a) Number of viable bacteria remaining (mean ± SD) on the root surface following treatment, (b) bioluminescence after 18 h incubation with plate reading every 15 min and (c) time to half‐maximum values. No statistically significant difference was observed between the test groups for any of the quantified parameters (n = 12)

Bacteria remaining in the furcation area after treatment were detached from the root surface and the CFUs were counted following 24 h incubation on tryptic soy agar plates (Figure 6). There was no statistically significant difference between the test groups. Negative control samples without bacteria inoculation were also plated and the CFUs counted following 24 h of incubation. The results for the negative control group consistently showed no bacterial growth (data not shown).

Regrowth of the bioluminescent bacteria detached from the furcations showed a similar pattern for the control group and the test groups (Figure 6(a)). Exponential growth phase started after 8 h incubation period with a peak luminescence value occurring after 14 h, after which a steady decline in luminescence was observed for all groups. Although the control group showed a higher and faster peak value, the difference was not statistically significant. In addition, the time to half‐maximum luminescence peak value (t_max/2_) was around 12 h for all four groups with no significant difference between the groups (Figure 6(b)).

## 4 DISCUSSION

The primary aim of this study was to investigate the use of porcine mandible blocks as an in vitro model for class II furcation defects. Once fully functional, the model is intended to be used for studying mechanical and biological properties of regenerative materials, the effect of periodontal treatment on root dentin and surrounding bone and antimicrobial agents used in the treatment of periodontal disease.

In order to use porcine teeth as a model for human teeth it was fundamental to establish how similar the two tissues were with regard to surface structure and response to mechanical force. The first step in development of the model was to compare the morphological and mechanical properties of the two tissues. Profilometer was used for surface roughness analysis on both porcine and human root dentin. The samples were polished prior to treatment to enable scanning by profilometer, effectively removing all cementum and the natural topography of the root surface. The volume of root dentin lost following debridement was not considered important to achieve the study aims and was excluded from analysis. Surface analysis data showed corresponding values between porcine and human teeth when analyzing the topographical pattern on both untreated control samples and following surface debridement as assessed by profilometer (Figure 6). This implies that porcine root dentin, within the limitations of this study, can be used as a model for human root dentin.

Porcine molars, like human molars, have multiple roots. Both porcine and human furcation have a large individual variation in root complex anatomy. The average horizontal depth of the furcation in the study sample was 3.2 ± 0.3 mm, which is comparable to a human class II furcation defect. The periodontal ligament (PDL) is a specialized connective tissue located between the root cementum and the alveolar bone. The narrowest part of the PDL in both humans and porcine is located around the furcation area and the width in this area are for both species is around 100 μm (Atriya, 2016; Beertsen, McCulloch, & Sodek, 1997; de Jong, Bakker, Everts, & Smit, 2017; Nanci & Bosshardt, 2006).

Porcine and human dentin have similar microstructural properties (Mlakar et al., 2014). There are differences in the organic and inorganic components. The hydroxyapatite crystals of both enamel and dentin are larger in humans than in porcine. It is reported that large crystal size is correlated with lower protein content (Ortiz‐Ruiz et al., 2018). Low protein content can cause hard tissues to become brittle, while a high protein content makes the tissues less wear resistant (Yilmaz, Schneider, & Swain, 2015). The difference in dentin composition did not appear to affect the surface morphology following different debridement methods in the present study (Figures 4 and 5). Porcine teeth were chosen as a model for human teeth due to similar genetic code and eating habits as humans (Genna, Annovazzi, Bonesi, Fogazzi, & Paganelli, 2007). Porcine teeth are readily available, cost effective and provide an ethically sound alternative for clinical research. There is a long tradition in medical research for using pigs, or miniature pigs, in various disease models including diseases of the oral cavity, salivary gland diseases and dental diseases such as periodontitis (Stembirek, Kyllar, Putnova, Stehlik, & Buchtova, 2012; Weaver, 1962). Although there are small differences in the anatomy and physiology of the periodontal tissues, enamel and dentin in porcine and humans, they are overall quite similar. For these reasons the miniature pig has been used extensively in periodontal research (Wang, Liu, Fang, & Shi, 2007). The present study follows a long tradition of using porcine as a model animal for humans. Porcine teeth should be considered a good analogue for human teeth.

In clinical practice, it is rare to work on 2D surfaces. In reality, the root dentin is presented on a 3D surface with large individual anatomical differences. The question then becomes: how can the surface inside a 3D structure be analyzed?

There are very few in vitro models that reproduce the access problem in periodontal treatment (Hagi et al., 2015), but none of them address the problem of access to a 3D surface such as a periodontal furcation area. The current model closely resembles the anatomy of an in vivo furcation defect which allow for a more natural simulation of the access problem in the furcation area.

The present model allowed for a detailed 3D view of the furcation area using SEM imaging. It gives a good basis for qualitative observations. The method was however not suited for extracting quantitative data. Data obtained from 2D surfaces using profilometer gives a good overview of quantitative differences between the different methods for creating surface changes, but also to compare human and porcine root dentin. When comparing the images from profilometer and SEM, the pattern appeared to have similar qualities. It was not possible within the current model to compare the two methods directly, mainly due to limitations related to measurement tools in the SEM. This presents a shortcoming in the current model. It was, however, a consistency in the images from profilometer and SEM that gives a good indication that the damage done on the flat sample (2D) is similar to those observed in the furcation defects (3D).

Having established that porcine teeth can be used as a model for human teeth and that analysis of root dentin surface on both 2D and 3D surfaces can be made, the next step was to test the model functionality. The ultimate purpose of the described model was to test in vitro clinically relevant problems such as periodontal regeneration. Keeping this in mind, a series of experiments was performed to assess the potential for analyzing a substance, in this case bacteria, inside the furcation area.

The purpose of bacterial growth in the present study was to show the functionality of the model. Initial testing using rhodamine B showed a successful seal between the root surface and the silicone material (Figure 1). This implied that a liquid placed within the furcation area would not penetrate beneath the silicone barrier. However, loss of surface tension was observed for a few of the samples during bacterial inoculation. As a consequence, these samples had less biofilm in the furcation area than average, most likely due to spreading of the seeded 200 μl bacteria droplet along the root surface in the gap between root and bone. The samples were immersed in 0.3 mM rhodamine B for 24 h before having root and the bone separated. Results showed a variance in the staining of the roots below the putty material sealing margin. The samples where the roots were not dislocated from their sockets during sample preparation showed less staining of the root surface beyond the exposed furcation area. This may also provide an explanation as to why some samples dried out during the initial inoculation. Even though the isolation technique presented in this study was considered adequate, the question remained as to how many, if any, bacteria were inoculated in the gap between the root surface and the surrounding bone. The sealing method worked, but not every time. Repositioning the root in its socket may result in a narrow gap between the root surface and the alveolar bone, while not lifting the root is likely to result in mechanical damage of the root surface during creation of the furcation defect in the alveolar bone. A potential solution to this problem could be to carefully seal the bone margin as well as the root surface, for example by using injectable low viscosity silicone impression material, to ensure complete sealing of the furcation area. Alternatively, the root surface at the furcation area may be temporarily protected from mechanical damage while preparing the furcation defect in the alveolar bone, which eliminates the need for dislocating the tooth from its socket and compromising the attachment of the periodontal ligament.

Different methods for removing the biofilm were applied to see if there were any differences in the bacterial load following simulated removal and, perhaps more importantly, to investigate if surface changes following removal could be analyzed inside the furcation area.

*S. epidermidis* Xen43 was used in this study to further investigate the remaining bacteria after incubation. *S. epidermidis* is not a periodontal pathogen. The purpose of this model was to explore how analysis of biofilm inside the furcation area could be performed. *S. epidermidis* is an excellent biofilm former and was primarily chosen for this reason (Paharik & Horswill, 2016). In addition to quantifying the colony forming units, the use of a bioluminescent bacterial strain allowed for real‐time quantification of bacterial growth, thus giving additional basis for evaluation of the remaining bacteria. While the results of the less accurate and laborious CFU counting experiments could be confirmed with the luminescence experiment, the use of constitutively bioluminescent bacteria strain also circumvents some of the problems associated with CFU counting, such as the potential of aggregation of bacteria or incomplete dispersal of biofilm during bath sonication. It is worth noting that negative control samples consistently showed no bacterial growth, indicating that the bacteria seen in the control and test groups were not present due to sample contamination.

Previous studies have shown that the luminescence correlates with bacterial growth until maximum luminescence (Wiedmer, Petersen, Lonn‐Stensrud, & Tiainen, 2017). The results from CFU counting and fluorescence readings showed no difference between control samples and the test groups. Within the limitations of the current model the biofilm could be assessed.

Future development of the furcation model should focus on improved sealing of the furcation area as this is the most important aspect to improve the functionality of the model. The use of human teeth should also be explored with a long‐term goal of a fully functional model to test regenerative material, antimicrobial agents and debridement methods. Alternative chemical and physical sterilization methods that better preserve the organic tissue matrix should be considered and tested in further development of the model. In future studies, bioluminescence alone could be used to measure the relative amount and viability of remaining bacteria in the furcation defect without the need for the less quantitative CFU counting method as good correlation was observed between the two methods.

## 5 CONCLUSIONS

The present study describes a novel method for studying class II furcation defects in a 3D model and represents the first step in the development of a method to standardize such in vitro investigations. Using porcine bone and teeth as an in vitro model may provide an accessible and cost‐effective method for assessing important clinical parameters, such as biofilm formation and reformation, surface roughness, possibly even antimicrobial agents and regeneration materials.

In conclusion

- The use of porcine mandibular blocks is a promising method to simulate class II furcation defect in vitro
- Porcine and human root dentin show similar surface morphology
- The model allowed for quantitative assessment of the root surface inside the furcation area
- Microbiological samples could be obtained and assessed

Within the limitations of this study, porcine teeth could be used as a model for human teeth. The present model simulated the anatomical conditions around class II furcation involved teeth. Further development of the current model is warranted.

## CONFLICT OF INTEREST

The authors declare no conflicts of interest.

## Supporting information


**Figure S1.** Illustration of the measurement of furcation height and depth from microCT sections. The furcation height was measured as the vertical distance from the cementoenamel junction (CEJ) to alveolar bone floor of the artificially created furcation defect, while the furcation depth was measured as the horizontal distance from the buccal root surface to the bottom of the furcation (n = 9).
**Figure S2.** Total volume of the created furcation defect was determined as the volume of missing tissues within the volume delimited by the defect wall and a planar surface extending vertically from the CEJ to the buccal bone surface at the bottom of created bony defect. The furcation defect was identified within the 3D microCT dataset (A) and a region of interest (ROI) containing the defect area in each of the image slices within the dataset was determined (B). A volume of interest (VOI) containing the entire furcation defect was then sectioned from the dataset by dynamic interpolation of the selected ROIs between the CEJ and buccal bone surface at the bottom of the bony defect (C).The grayscale images were then binarised by applying a threshold that excluded all hard tissue within the VOI to created a 3D object corresponding to the furcation defect volume (D).
**Figure S3.** Morphological features of the created class II furcations defects. The average height and depth of the furcation defect were measured from the microCT image section to be 3.1 ± 0.5 mm and 3.4 ± 0.8 mm, respectively. The depth measurements corroborate the clinical furcation depth measurements performed using a physical probe (3.2 ± 0.3 mm). The average volume of the created furcation defects was 26.8 ± 5.7 mm^3^, including the entire buccal osteomy. Boxplots show 25/75 percentiles, minima, maxima, and median values (n = 9).

## ENDNOTES

^1^
Exact Technologies, Norderstedt, Germany

^2^
Ra 0.21, Hager and Meisinger GmbH, Neuss, Germany

^3^
Technovit, Kulzer GmbH, Hanau, Germany

^4^
Provil Novo, Kulzer GmbH, Hanau, Germany

^5^
Skyscan 1,172, Skyscan, Kontich, Belgium

^6^
Bruker, Billerica, MA

^7^
TM‐3030, Hitachi, Tokyo, Japan

^8^
S neox, Sensofar Group, Barcelona, Spain

^9^
Magnum Bond rapid, Magnum Industries, Chennai, India

^10^
Buehler‐Met, Lake Bluff, IL

^11^
American Eagle Instruments Inc., Missoula, MT

^12^
Foss Viking AS, Fetsund, Norway

^13^
Satelec Aceton, Merignac, France

^14^
Biophotometer, Eppendorf, Hamburg, Germany

^15^
Thermo Fisher Scientific, Waltham, MA

^16^
OptiPlate‐96, PerkinElmer, Waltham, MA

^17^
TopSeal A‐Plus, PerkinElmer, Waltham, MA

^18^
Synergy HT, BioTek Instruments, Inc., Winooski, VT

^19^
108auto, Cressington, Watford, England

^20^
TM‐3030, Hitachi, Tokyo, Japan

^21^
Adobe Photoshop CC, Adobe Systems, San Jose, CA
