# Supplementary material for: Development and initial testing of an in vitro model simulating class II furcation defects
Source: Clin Exp Dent Res. 2020 Dec 6;7(2):179–88. doi: 10.1002/cre2.346 (PMC8019757; doi:10.1002/cre2.346)
Supplement: Supplementary file 1 — Figure S1. Illustration of the measurement of furcation height and depth from microCT sections. The furcation height was measured as the vertical distance from the cementoenamel junction (CEJ) to alveolar bone floor of the artificially created furcation defect, while the furcation depth was measured as the horizontal distance from the buccal root surface to the bottom of the furcation (n = 9). Figure S2. Total volume of the created furcation defect was determined as the volume of missing tissues within the volume delimited by the defect wall and a planar surface extending vertically from the CEJ to the buccal bone surface at the bottom of created bony defect. The furcation defect was identified within the 3D microCT dataset (A) and a region of interest (ROI) containing the defect area in each of the image slices within the dataset was determined (B). A volume of interest (VOI) containing the entire furcation defect was then sectioned from the dataset by dynamic interpolation of the selected ROIs between the CEJ and buccal bone surface at the bottom of the bony defect (C).The grayscale images were then binarised by applying a threshold that excluded all hard tissue within the VOI to created a 3D object corresponding to the furcation defect volume (D). Figure S3. Morphological features of the created class II furcations defects. The average height and depth of the furcation defect were measured from the microCT image section to be 3.1 ± 0.5 mm and 3.4 ± 0.8 mm, respectively. The depth measurements corroborate the clinical furcation depth measurements performed using a physical probe (3.2 ± 0.3 mm). The average volume of the created furcation defects was 26.8 ± 5.7 mm3, including the entire buccal osteomy. Boxplots show 25/75 percentiles, minima, maxima, and median values (n = 9). [file CRE2-7-179-s001.pdf]

Development and initial testing of an *in vitro* model simulating class II furcation defects

Jørgen Hugo, DDS*,†, Odd Carsten Koldsland, PhD, associate professor*, Anne Merete Aass, PhD, professor*, Hanna Tiainen† , PhD, associate professor.

*Department of Periodontology and †Department of Biomaterials, Institute of Clinical Dentistry, University of Oslo, Norway.

Supporting Information

Table of contents:

Figure S1.. 2
Figure S2.. 3
Figure S3.. 4

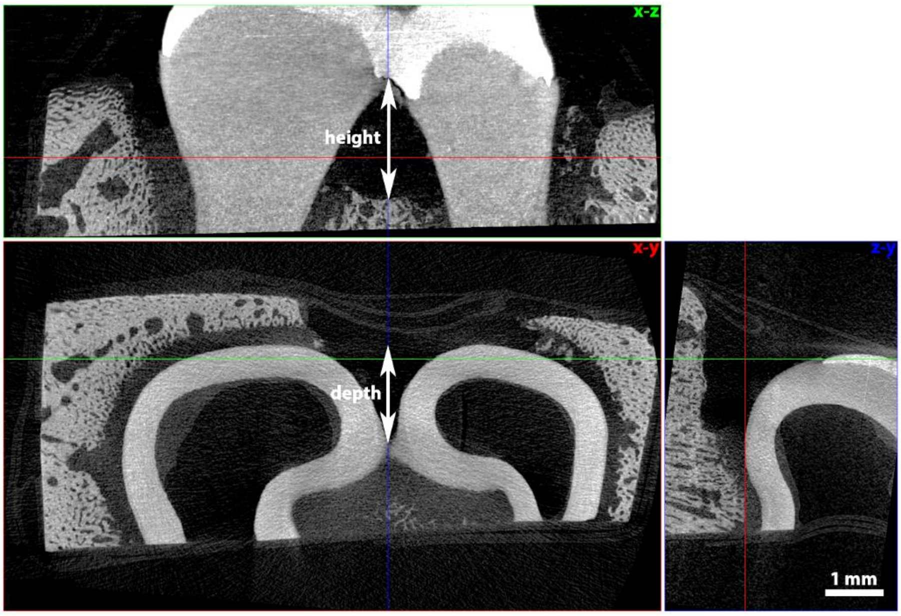

Figure S1: Illustration of the measurement of furcation height and depth from microCT sections. The furcation height was measured as the vertical distance from the cementoenamel junction (CEJ) to alveolar bone floor of the artificially created furcation defect, while the furcation depth was measured as the horizontal distance from the buccal root surface to the bottom of the furcation (n = 9).

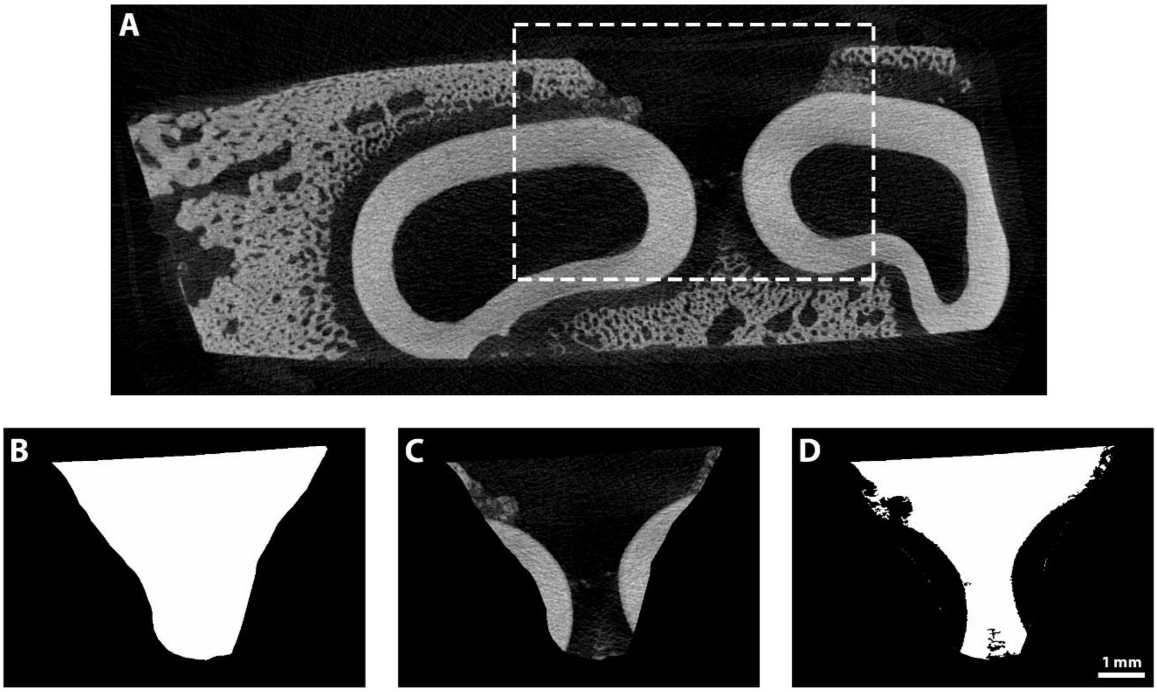

Figure S2: Total volume of the created furcation defect was determined as the volume of missing tissues within the volume delimited by the defect wall and a planar surface extending vertically from the CEJ to the buccal bone surface at the bottom of created bony defect. The furcation defect was identified within the 3D microCT dataset **(A)** and a region of interest (ROI) containing the defect area in each of the image slices within the dataset was determined **(B)**. A volume of interest (VOI) containing the entire furcation defect was then sectioned from the dataset by dynamic interpolation of the selected ROIs between the CEJ and buccal bone surface at the bottom of the bony defect **(C)**.The grayscale images were then binarised by applying a threshold that excluded all hard tissue within the VOI to created a 3D object corresponding to the furcation defect volume **(D)**.

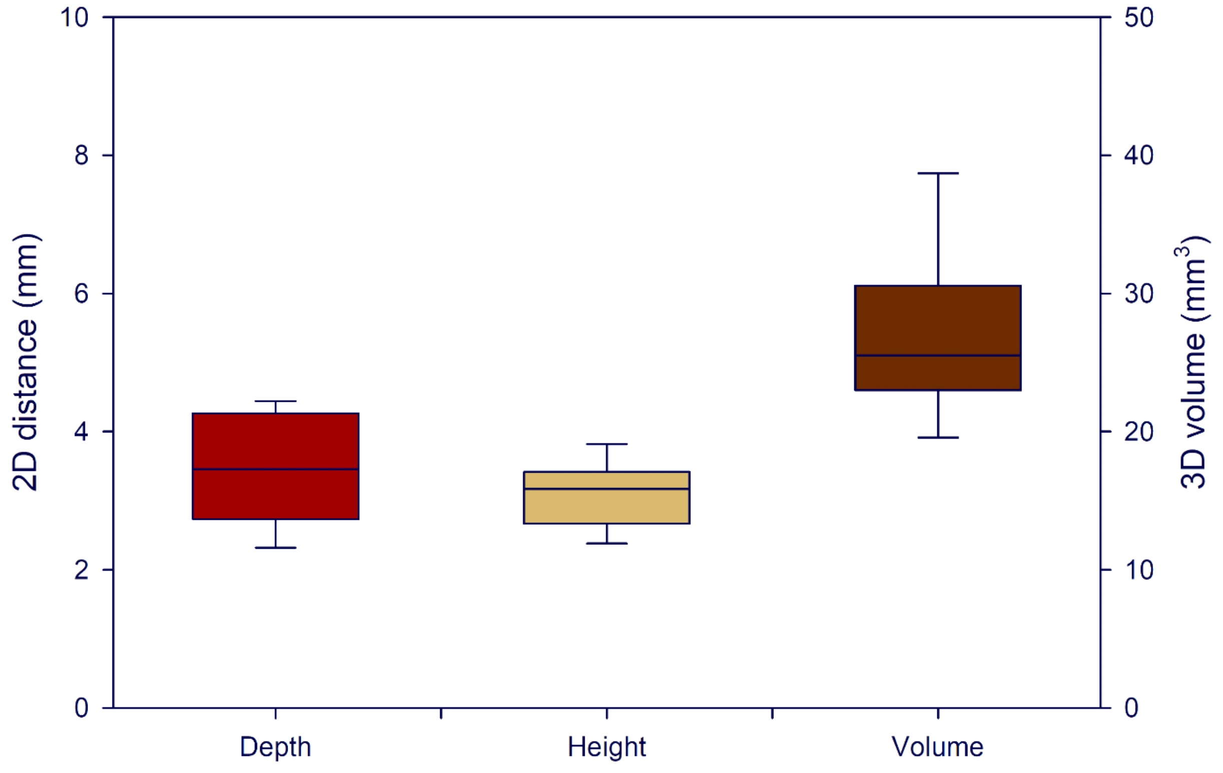

Figure S3: Morphological features of the created class II furcations defects. The average height and depth of the furcation defect were measured from the microCT image section to be 3.1 ± 0.5 mm and 3.4 ± 0.8 mm, respectively. The depth measurements corroborate the clinical furcation depth measurements performed using a physical probe (3.2 ± 0.3 mm). The average volume of the created furcation defects was 26.8 ± 5.7 mm^3, including the entire buccal osteomy. Boxplots show 25/75 percentiles, minima, maxima, and median values (n = 9).
